# Lung cancer costs by treatment strategy and phase of care among patients enrolled in Medicare

**DOI:** 10.1002/cam4.1896

**Published:** 2018-12-21

**Authors:** Deirdre F. Sheehan, Steven D. Criss, Yufan Chen, Andrew Eckel, Lauren Palazzo, Angela C. Tramontano, Chin Hur, Lauren E. Cipriano, Chung Yin Kong

**Affiliations:** ^1^ Institute for Technology Assessment Massachusetts General Hospital Boston Massachusetts; ^2^ Department of Medicine Harvard Medical School Boston Massachusetts; ^3^ Ivey Business School Western University London Ontario Canada; ^4^ Department of Radiology Harvard Medical School Boston Massachusetts

**Keywords:** healthcare costs, lung cancer, phase of care, SEER‐Medicare, treatment

## Abstract

**Background:**

We studied trends in lung cancer treatment cost over time by phase of care, treatment strategy, age, stage at diagnosis, and histology.

**Methods:**

Using the Surveillance, Epidemiology, and End Results (SEER)‐Medicare database for years 1998‐2013, we allocated total and patient‐liability costs into the following phases of care for 145 988 lung cancer patients: prediagnosis, staging, surgery, initial, continuing, and terminal. Patients served as self‐controls to determine cancer‐attributable costs based on individual precancer diagnosis healthcare costs. We fit linear regression models to determine cost by age and calendar year for each stage at diagnosis, histology, and treatment strategy and presented all costs in 2017 US dollars.

**Results:**

Monthly healthcare costs prior to lung cancer diagnosis were $861 for a 70 years old in 2017 and rose by an average of $17 per year (*P* < 0.001). Surgery in 2017 cost $30 096, decreasing by $257 per year (*P* = 0.007). Chemotherapy and radiation costs remained stable or increased for most stage and histology groups, ranging from $4242 to $8287 per month during the initial six months of care. Costs during the final six months of life decreased for those who died of lung cancer or other causes.

**Conclusions:**

Cost‐effectiveness analyses of lung cancer control interventions in the United States have been using outdated and incomplete treatment cost estimates. Our cost estimates enable updated cost‐effectiveness analyses to determine the benefit of lung cancer control from a health economics point of view.

## 1 INTRODUCTION

Lung cancer is the leading cause of cancer death in the United States and the second most commonly diagnosed cancer for both men and women.^1^, ^2^ The American Cancer Society estimates that in 2017, there were 222 500 new lung cancer cases diagnosed in the United States and 155 870 lung cancer deaths.^2^ There is an urgent need to reduce the disease burden of lung cancer, and the prevention and treatment of lung cancer have recently experienced dramatic changes. For example, based on the observed 20% lung cancer mortality reduction in the National Lung Screening Trial (NLST), the Centers for Medicare and Medicaid Services (CMS) announced in 2015 that Medicare would cover the cost of lung cancer screening with computed tomography (CT) for individuals meeting certain age and smoking history criteria.^3^, ^4^

While new cancer control interventions can mitigate the health burdens of lung cancer, it is important to understand these interventions’ economic burdens on society. However, many of the cost‐effectiveness analyses (CEAs) that have been performed to understand the economic value of new lung cancer screening recommendations and treatment options use either incomplete or outdated cost estimates.^5^, ^6^, ^7^, ^8^, ^9^, ^10^ For researchers to accurately assess the cost‐effectiveness of lung cancer control, there is a pressing need to establish cancer treatment cost estimates that are both up to date and comprehensive with respect to multiple distinct phases of cancer care.

To ensure accurate cost estimates for future CEA studies of lung cancer control, we estimated lung cancer treatment costs over time by age, stage at diagnosis, histology, and treatment strategy for patients over the age of 65, the eligibility age of Medicare. We calculated costs associated with patient‐level lung cancer treatment using the SEER‐Medicare database for years 1998‐2013 and adjusted costs to 2017 US dollars. Additionally, for patients who enrolled in Medicare Part D, we investigated prescription drug costs before and after lung cancer diagnosis for years 2007‐2013. Using these patient‐level cost data, we assessed trends, by phase of care, in cost of lung cancer treatment for patients enrolled in Medicare in the United States.

## 2 MATERIALS AND METHODS

### 2.1 SEER‐Medicare data

Our analysis was performed using the Surveillance, Epidemiology, and End Results (SEER)‐Medicare database for years 1998‐2013. SEER is a resource of the National Cancer Institute that compiles clinical, demographic, and cause of death information from 17 cancer registries across the United States, representing about 28% of the US population.^11^ Medicare provides health insurance for 97% of Americans age 65 and older, nearly all of whom have Part A and Part B coverage.^12^ SEER‐Medicare represents a linkage of data for people diagnosed with cancer in any of the SEER geographic regions who were also enrolled in Medicare; 93% of those aged 65 and older in the SEER files are linked to the Medicare enrollment file.^12^ A detailed description of SEER‐Medicare can be found at https://healthcaredelivery.cancer.gov/seermedicare/.

Our cohort included Medicare beneficiaries 65 years or older who were diagnosed with American Joint Committee on Cancer (AJCC) stage I‐IV lung cancer between 2000 and 2011. Patients were excluded if lung cancer stage was not recorded, if they had previous or subsequent cancer diagnoses other than lung cancer, if they were not continuously enrolled in both Medicare Part A and Part B coverage during the period of 15 months prior to cancer diagnosis through death or the end of 2013, if they received Medicare benefits because of disability or end‐stage renal disease, if they enrolled in managed care at any time during the study period, if month of diagnosis was unknown, if diagnosis was made at autopsy, or if the date of death recorded in the Medicare database differed from that of the SEER database by more than three months.

### 2.2 Treatment strategies

Codes used to define chemotherapy, radiation, and surgery can be found in Appendix S1 (Supplementary Methods). For patients diagnosed with AJCC stages I through III lung cancer, we defined treatment strategies based on treatment(s) each patient received during the period of two months prior to lung cancer diagnosis through six months after diagnosis. For patients diagnosed with stage IV lung cancer, treatment groups were defined by treatment(s) ever received. We also report costs of best supportive care for patients whose cancers were not actively treated with surgery, chemotherapy, or radiation. Non‐small‐cell lung cancer (NSCLC) is reported as AJCC stages I‐IV, and small‐cell lung cancer (SCLC) stage is reported as limited (AJCC stages I‐III) or extensive (AJCC stage IV).

### 2.3 Phases of care

The prediagnosis phase—defined as the one‐year period ending three months prior to lung cancer diagnosis—was used to estimate what that patient's healthcare spending would have been had the patient not developed lung cancer. The three months prior to diagnosis were excluded from cost calculations so that the average baseline costs would not be influenced by treatments given to symptomatic patients not yet diagnosed with lung cancer. Monthly costs relative to diagnosis date can be found in Appendix S2 (Figure S1).

A patient who received only nonsurgical treatment(s) or best supportive care has a one‐month staging phase beginning on the date of lung cancer diagnosis. In practice, staging can occur over a period longer than one month for some patients; the one‐month staging phase is based on clinical practice at our institution. A patient treated with surgery has a one‐month surgery phase beginning on the date of surgery. Since many patients (38.0%) received surgery within one month of diagnosis, the inconsistent amount of time between diagnosis and surgery dates was excluded. The staging or surgery phase is followed by a six‐month initial phase, a continuing phase that varies in length depending on how long the patient lived following diagnosis, and, if a patient died before 31 December 2013, a six‐month terminal phase ending on the date of death. We saw a clear rise in costs around 6 months before death; further explanation of our decision to use of a six‐month terminal phase can be found in Appendix S2 (Figure S2). Costs are first allocated to the terminal phase, followed by the staging or surgery phase, the initial phase, and the continuing phase. Patients who survived fewer than 30 days following surgery are defined as operative deaths.

### 2.4 Cancer‐attributable costs

Costs were defined as the sum of Medicare reimbursements (payments from Medicare to the service provider), coinsurance reimbursements (payments from a coinsurer to the service provider), and deductibles and copayments billed to patients. We determined total and patient‐liability costs, although within SEER‐Medicare data, patient‐liability costs paid out of pocket at the time of service cannot be differentiated from those paid by a purchased Medigap policy—insurance sold by private companies to help cover coinsurance, copayment, and deductible costs.^13^

Each patient's cancer‐attributable costs were determined by subtracting the patient's own mean monthly prediagnosis phase costs from the mean monthly costs during treatment phases. The self‐control method enabled us to control for the prior presence of comorbid conditions and health‐related behaviors that may be correlated with healthcare costs, but are not included in SEER‐Medicare data, such as smoking status.^14^ Cancer‐attributable costs incorporate not only the direct cost of cancer treatment, but also other healthcare costs incurred during phases of treatment beyond the average prediagnosis healthcare costs. A sensitivity analysis to determine the effect that increasing prediagnosis baseline healthcare costs, due to comorbidity or other causes, would have on cancer‐attributable costs was also conducted (Figure S3 and Table S1 in Appendix S2).

Payments were converted to constant 2017 US dollars by adjusting Part A claims using the CMS Prospective Payment System Hospital Price Index and Part B claims using the Medicare Economic Index.^15^, ^16^ All costs are presented in inflation‐adjusted US dollars.

### 2.5 Prescription drug costs

We analyzed prescription drug costs, including patient‐liability costs, for the subset of patients who were consistently enrolled in Medicare Part D, also known as the Medicare prescription drug benefit, during 2007‐2013 from lung cancer diagnosis through death or 31 December 2013. We additionally determined average costs for those treated with erlotinib hydrochloride (Tarceva®, OSI Pharmaceuticals, Inc, Melville, NY and Genentech, Inc, San Francisco, CA), a drug currently approved for first‐line treatment of metastatic NSCLC patients whose tumors have certain epidermal growth factor receptor (EGFR) mutations.^17^, ^18^, ^19^, ^20^ Consistent with previously published work,^21^ Part D claims were converted to 2017 US dollars using the pharmaceutical and medical components of the Producer Price Index.^22^

### 2.6 Statistical analysis

We calculated total, patient‐liability, and cancer‐attributable monthly costs for each patient. Multiple linear regression models were fit to estimate population average total, patient‐liability, and cancer‐attributable monthly costs for each phase. Separate models were fit for each phase, stage at diagnosis, histology, and treatment strategy. Models for monthly prescription drug costs were fit for each phase and treatment strategy. Treatment strategy costs are not shown if less than 10% of patients within a stage/histology group received that treatment; best supportive care costs are shown for all groups (see Tables S2 and S3 in Appendix S2 for percentage of patients treated with each strategy, by stage and histology). Calendar year, age, and an interaction term were included as independent terms in the models and were dropped, using backward stepwise selection, until all terms were significant at the *α* = 0.05 level. Parameter estimates for all models can be found in Appendix S3(Regression Parameters). All analyses were done using SAS 9.4 (Cary, NC). For illustrative purposes and ease of comparisons across patient groups and phases of care, average costs are presented for a representative patient at age 70 in 2017, unless otherwise noted. If neither age nor year was significantly correlated with cost, mean costs for patients in that group are shown.

## 3 RESULTS

A total of 145 988 lung cancer patients were included in the analysis (Table 1). Over half of the patients (51.9%) were diagnosed between 70 and 79 years of age, and the median age of diagnosis was 75. Most patients (73.5%) were diagnosed with NSCLC; 26.5% were diagnosed with SCLC. Of the 133 447 patients (91.4%) who died during the study period, 77.5% died of lung cancer. The average time from diagnosis to death was 14.0 months; average survival for those who died of lung cancer was 11.1 months. Many patients (64%) did not have a continuing phase, meaning they lived 13 months or fewer following diagnosis or surgery. About half (49%) did not have an initial or a continuing phase, meaning they lived seven months or fewer following diagnosis or surgery.

**Table 1:** Description of 145 988 patients included in the analysis

	Number	Percent
Male	75 319	51.6
Year of diagnosis		
2000	11 902	8.15
2001	12 511	8.57
2002	12 534	8.59
2003	13 225	9.06
2004	12 516	8.57
2005	12 523	8.58
2006	12 308	8.43
2007	12 103	8.29
2008	11 860	8.12
2009	11 897	8.15
2010	11 543	7.91
2011	11 066	7.58
Age at diagnosis		
65‐69 y	28 375	19.44
70‐74 y	38 900	26.65
75‐79 y	36 981	25.33
80+ y	41 732	28.59
Histologic type and stage at diagnosis		
Stage I/II NSCLC	32 848	22.5
Stage III NSCLC	30 422	20.8
Stage IV NSCLC	43 981	30.1
Limited stage SCLC	16 969	11.6
Extensive stage SCLC	21 768	14.9
Treatment strategy		
Best supportive care	39 865	27.3
Surgery^a^	18 734	12.8
Chemotherapy	13 312	9.1
Radiation	32 108	22.0
Chemotherapy and radiation	32 572	22.3
Cause of death^b^		
Lung cancer	103 459	77.5
Operative^c^	1431	1.1
All other causes	28 557	21.4

NSCLC, non‐small‐cell lung cancer; SCLC, small‐cell lung cancer.

a 2.9% of patients received surgery and radiation, 1.7% of patients received surgery and chemotherapy, and 1.8% of patients received surgery, radiation, and chemotherapy.

b Percentages represent proportions among the 133 447 (91.4%) total deaths during the study period.

c Operative death is defined as death within 30 days of surgery to remove lung cancer.

### 3.1 Costs during the prediagnosis and staging phases

Monthly healthcare costs during the prediagnosis phase were subtracted from mean monthly costs during treatment phases to determine cancer‐attributable costs. For a patient diagnosed at age 70 in 2017, average costs per month during the prediagnosis phase were $861 (95% confidence interval [CI], $826 to $896), of which the patient was responsible for $84 (95% CI, $81 to $88) (see Table S4 in Appendix S2). Prediagnosis costs significantly increased with year of diagnosis (*P* < 0.0001) and age of diagnosis (*P* < 0.0001). Average monthly costs during the three months immediately prior to diagnosis, which was excluded from the prediagnosis phase, were significantly different (*P* < 0.001) from average monthly costs during the one‐year period ending three months prior to diagnosis.

For patients who did not receive surgery, costs during the month of staging range from $6670 (95% CI, $6014 to $7327) to $13 608 (95% CI, $10 399 to $16 818) (see Table S5 in Appendix S2). Staging costs among those who received chemotherapy, radiation, or both tended to be lower for older patients. Among patients who received best supportive care, staging costs tended to be higher for older patients. Patient‐liability costs during the staging phase ranged from $661 (95% CI, $379 to $944) to $1383 (95% CI, $1267 to $1499. Treatment regimens beginning within 30 days of diagnosis may have contributed to differences in staging phase costs.

### 3.2 Cost of initial cancer treatment

For a patient who received surgery at age 70 in 2017, average costs during the month beginning on the date of surgery were $30 096 (95% CI, $27 855 to $32 337); the patient was liable for $1738 (95% CI, $1625 to $1852). Although total cost decreased by $257 each calendar year (*P* = 0.007), patient liability increased by $22 each year (*P* < 0.0001). Cost of surgery did not change significantly with age at diagnosis.

**Figure 1:**
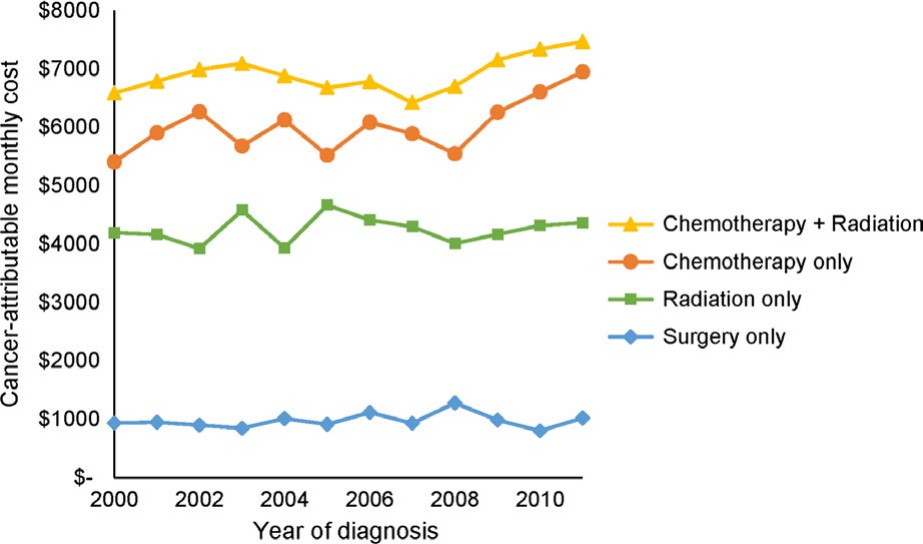
Average monthly cancer‐attributable costs are presented for the initial phase, by treatment strategy. Costs ranged from $802 per month for patients who received surgery to $7469 per month for patients who received chemotherapy plus radiation

Average monthly cancer‐attributable costs during the six‐month initial treatment phase—which, for patients who received surgery, begins 30 days after the date of surgery—vary widely by treatment strategy (Figure 1). Results from the linear regression models for monthly total costs and cancer‐attributable costs are shown by stage, histology, and treatment strategy in Table 2. Cancer‐attributable costs for a 70‐year‐old patient who received chemotherapy, radiation, or both ranged from $4242 (95% CI, $3709 to $4775) to $8287 (95% CI, $7735 to $8839) per month during the initial phase, while costs for those who received surgery were $828 (95% CI, $741 to $916) per month. The average cancer‐attributable cost of best supportive care ranged from $1672 (95% CI, $1513 to $1830) to $2991 (95% CI, $2528 to $3455) per month. Among patients who were treated with chemotherapy only, initial phase cancer‐attributable costs ranged from $6187 (95% CI, $5863 to $6510) to $8146 (95% CI, $7208 to $9083) per month and, for NSCLC patients, increased over time. SCLC patients’ chemotherapy costs did not change over time. For patients who received radiation only, costs significantly decreased with age in most groups and increased over time for stage I‐III NSCLC. Among patients who received both chemotherapy and radiation, costs decreased with age, increasing over time for stage IV NSCLC patients only. Patient‐liability costs during the initial phase ranged from $152 (95% CI, $144 to $159) to $1200 (95% CI, $1073 to $1327) and were highest for those who received chemotherapy or chemotherapy plus radiation.

**Table 2:** Significant predictors of cost during the initial phase and monthly costs for a patient aged 70 in 2017 by histology, stage at diagnosis, and treatment strategy^a^

	N (%)	Total cost	95% CI	Patient‐liability cost	95% CI	Cancer‐attributable cost	95% CI	Predictors	
								Year	Age
NSCLC									
Stages I and II	26 959								
Best supportive care	3913 (14.5)	$3146	$2595 to $3697	$247	$232 to $263	$1672	$1513 to $1830		
Surgery	13 872 (51.5)	$1676	$1450 to $1901	$152	$144 to $159	$828	$741 to $916		**+**
Radiation	3453 (12.8)	$5921	$5343 to $6499	$736	$655 to $816	$4242	$3709 to $4775	**+**	
Stage III	16 282								
Best supportive care	3335 (20.5)	$2931	$2775 to $3087	$214	$151 to $277	$2168	$2009 to $2327		
Surgery	1577 (9.7)	$3312	$2360 to $4263	$462	$284 to $639	$2663	$1706 to $3621		
Radiation	2973 (18.3)	$7146	$6286 to $8005	$742	$618 to $866	$6037	$5156 to $6919	**+**	−
Chemotherapy	1902 (11.7)	$8679	$7757 to $9600	$1200	$1072 to $1327	$8146	$7208 to $9083	**+**	−
Chemotherapy and radiation	4509 (27.7)	$7946	$7723 to $8168	$1149	$1064 to $1233	$7496	$7270 to $7723		−
Stage IV	13 583								
Best supportive care	1830 (13.5)	$3912	$3457 to $4366	$326	$292 to $361	$2991	$2528 to $3455		−
Radiation	2349 (17.3)	$6196	$5740 to $6652	$529	$403 to $655	$5400	$4942 to $5856		−
Chemotherapy	2050 (15.1)	$7199	$6229 to $8168	$941	$892 to $990	$6825	$5825 to $7825	**+**	
Chemotherapy and radiation	6283 (46.3)	$8739	$8191 to $9289	$1189	$1160 to $1217	$8287	$7735 to $8840	**+**	−
SCLC									
Limited stage	10 331								
Best supportive care	1581 (15.3)	$2574	$2365 to $2783	$297	$270 to $325	$1857	$1644 to $2071		
Radiation	1871 (18.1)	$5528	$5146 to $5910	$581	$438 to $724	$4681	$4276 to $5087		−
Chemotherapy	1523 (14.7)	$6862	$6423 to $7301	$995	$955 to $1035	$6347	$5906 to $6787		
Chemotherapy and radiation	4057 (39.3)	$8597	$7948 to $9246	$1175	$1083 to $1267	$7364	$7158 to $7570		−
Extensive stage	7591								
Best supportive care	445 (5.9)	$3094	$2632 to $3556	$523	$89 to $956	$2367	$1895 to $2839		
Chemotherapy	1364 (18.0)	$6713	$6395 to $7032	$768	$560 to $977	$6187	$5863 to $6510		
Chemotherapy and radiation	4917 (64.8)	$7449	$7227 to $7672	$1059	$967 to $1150	$7050	$6826 to $7274		−

NSCLC, non‐small‐cell lung cancer; SCLC, small‐cell lung cancer

a The directions of significant predictors are shown for linear regression models of cancer‐attributable costs. A positive (+) symbol indicates that the covariate in the regression model has a parameter estimate greater than 0, while a negative (−) symbol indicates that the parameter estimate is less than 0. With the exception of best supportive care costs, treatment strategy costs are not shown if less than 10% of patients within a stage/histology group received that treatment. Coefficients for the fitted regressions are presented in Appendix S3: regression parameters.

### 3.3 Costs during the continuing and terminal phases

**Figure 2:**
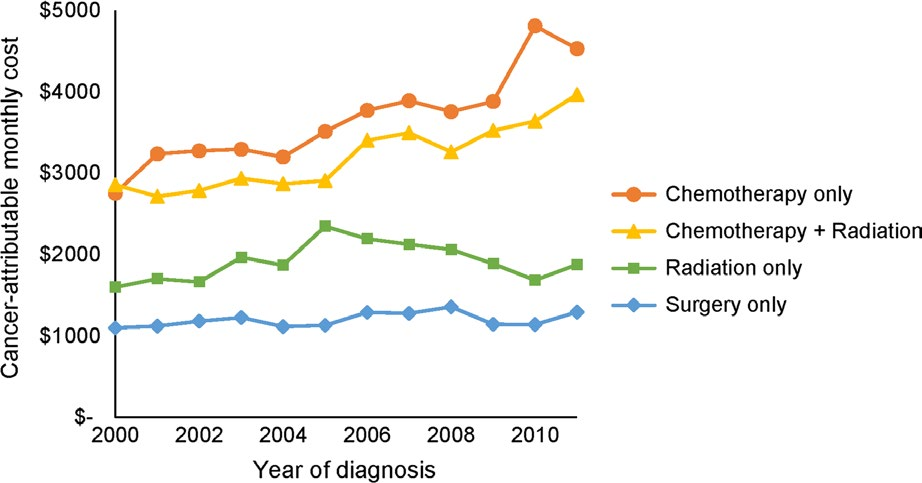
Average monthly cancer‐attributable costs are presented for the continuing phase, by treatment strategy. Costs ranged from $1100 per month for patients who received surgery to $4809 per month for patients who were treated with chemotherapy

Among patients with a continuing phase of at least 30 days, the median length of the continuing phase was 21.8 months. Compared to the initial phase, average monthly cancer‐attributable costs during the continuing phase varied less by treatment strategy (Figure 2). Costs were generally lower than those of the initial phase, ranging from $1269 (95% CI, $1049 to $1490) in stage IV NSCLC patients who received best supportive care to $5756 (95% CI, $4937 to $6574) in stage III NSCLC patients who received chemotherapy (Table 3). Costs during the continuing phase remained stable or increased during the study period and were either unaffected by age or decreased for older patients. Patient‐liability costs were generally lower than those of the staging and initial phases.

**Table 3:** Significant predictors of cost during the continuing phase and monthly costs for a patient aged 70 in 2017 by histology, stage at diagnosis, and treatment strategy^a^

	N (%)	Total cost	95% CI	Patient‐liability cost	95% CI	Cancer‐attributable cost	95% CI	Predictors	
								Year	Age
NSCLC									
Stages I and II	23 712								
Best supportive care	3199 (13.5)	$2689	$2359 to $3019	$279	$242 to $315	$1763	$1421 to $2105	+	
Surgery	13 197 (55.7)	$1869	$1763 to $1976	$242	$228 to $255	$1343	$1229 to $1458	+	
Radiation	2586 (10.9)	$3105	$2741 to $3469	$367	$327 to $407	$1673	$1490 to $1856		−
Stage III	10 960								
Best supportive care	2255 (20.6)	$3817	$3333 to $4301	$497	$433 to $560	$3263	$2752 to $3774	+	−
Surgery	1395 (12.7)	$2761	$2341 to $3181	$357	$309 to $405	$2167	$1745 to $2589	+	
Radiation	1813 (16.5)	$3909	$3309 to $4508	$516	$447 to $586	$3067	$2450 to $3683	+	−
Chemotherapy	1184 (10.8)	$6315	$5525 to $7103	$896	$781 to $1011	$5756	$4937 to $6574	+	−
Chemotherapy and radiation	2738 (25.0)	$2759	$2624 to $2895	$401	$382 to $421	$2352	$2214 to $2490		
Stage IV	7443								
Best supportive care	893 (12.0)	$2983	$2309 to $3657	$188	$162 to $215	$1269	$1049 to $1490		
Radiation	1091 (14.7)	$3084	$2778 to $3389	$348	$309 to $387	$2201	$1884 to $2518		−
Chemotherapy	1124 (15.1)	$5389	$4645 to $6133	$816	$705 to $926	$5104	$4301 to $5908	+	−
Chemotherapy and radiation^b^	3512 (47.2)	$6181	$5717 to $6644	$970	$895 to $1045	$5738	$5270 to $5908	+	
SCLC									
Limited stage	6928								
Best supportive care	1154 (16.7)	$2471	$2298 to $2643	$359	$328 to $390	$1848	$1664 to $2032		
Radiation	1280 (18.5)	$3586	$2928 to $4243	$384	$353 to $416	$1867	$1661 to $2073		
Chemotherapy	868 (12.5)	$5279	$4296 to $6262	$637	$588 to $685	$4821	$3831 to $5811	+	−
Chemotherapy and radiation	2508 (36.2)	$3010	$2582 to $3438	$400	$382 to $418	$2338	$2208 to $2469		−
Extensive stage	3476								
Best supportive care	198 (5.7)	$2097	$1686 to $2508	$145	$61 to $230	$1276	$800 to $1751		
Chemotherapy	561 (16.1)	$3822	$3509 to $4136	$647	$588 to $707	$3297	$2963 to $3632		
Chemotherapy and radiation	2316 (66.6)	$4970	$4376 to $5563	$704	$673 to $735	$4444	$3836 to $5052	+	−

a The directions of significant predictors are shown for linear regression models of cancer‐attributable costs. A positive (+) symbol indicates that the covariate in the regression model has a parameter estimate greater than 0, while a negative (−) symbol indicates that the parameter estimate is less than 0. With the exception of best supportive care costs, treatment strategy costs are not shown if less than 10% of patients within a stage/histology group received that treatment. Coefficients for the fitted regressions are presented in Appendix S3.

b The interaction term between year and age was significant in the model for continuing phase stage IV NSCLC patients who received chemotherapy and radiation. The coefficient of the interaction term was negative. Costs increased with year. Age was kept in the model although it was not significant (*P* = 0.37) to ensure that the model's terms were hierarchically well‐formulated.

Total monthly costs during the terminal phase—the six months preceding a patient's death—are shown for patients who died of lung cancer or other causes (Table S6 in Appendix S2). Total costs decrease with year and age in both models, although the rate at which costs decrease over time is faster for older patients than for younger patients, among whom costs remain somewhat stable. Monthly costs for nonlung cancer deaths also decreased at a faster rate over time than costs for lung cancer deaths. In 2010, average monthly costs during the terminal phase were similar between those who died of lung cancer and other causes—$13 426 (95% CI, $13 242 to $13 610) vs $13 840 (95% CI, $13 471 to $14 208) for a 70 years old—but by 2017, the average monthly costs of the terminal phase were lower for nonlung cancer deaths—$12 987 (95% CI, $12 586 to $13 389) vs $10 266 (95% CI, $9645 to $10 887) for a 70 years old. A patient who died in 2017 would be liable for a comparable, though higher, cost during the six months preceding a lung cancer death compared to a nonlung cancer death—$1161 (95% CI, $1133 to $1190) vs $919 (95% CI, $862 to $976) for a 70 years old. Patient‐liability costs decreased over time.

### 3.4 Prescription drug costs

For the 32 165 patients in our study who were enrolled in Medicare Part D during 2007‐2013, average prescription drug costs increased over time. Prescription drug costs can be found in Appendix S2 (Table S7). Total average costs were highest during the terminal phase ($1050 (95% CI, $954 to $1146) per month for a patient age 70 in 2017). Patients were liable for the highest prescription drug costs during the staging phase ($184 (95% CI, $164 to $203) for a patient age 70 in 2017).

Of 3100 patients who were enrolled in Medicare Part D and were prescribed a targeted therapy drug, 3058 (98.7%) received erlotinib. The mean monthly cost of erlotinib during 2007‐2013 was highest during the terminal phase, at $1844 (95% CI, $1777 to $1912) per month; the patient was responsible for $252 (95% CI, $236 to $268) per month. Monthly costs of erlotinib during other phases of care can be found in Appendix S2 (Table S8).

## 4 DISCUSSION

Prior to lung cancer diagnosis, our results indicate that baseline healthcare costs increased and patients had greater liability for these costs. Staging costs ranged from $6670 to $13 608 and were relatively stable over time. For early‐stage patients who receive surgery, costs during the month of surgery have been decreasing and costs during the initial and continuing phases have been considerably lower than those of chemotherapy, radiation, or both. Costs of chemotherapy and radiation for late‐stage patients have increased, while costs during a lung cancer patient's six‐month terminal phase decreased over time both for patients who died of lung cancer and for patients who died of other causes, although costs decreased more quickly over the study period for nonlung cancer deaths compared with lung cancer deaths. Monthly prescription drug costs were highest during the terminal phase.

Lung cancer treatment costs have been previously published for patients diagnosed between 1992 and 2002 in the United States, using SEER‐Medicare data and a self‐control method with similar phase of care definitions.^10^ That analysis and ours found that costs are changing over time for some patient groups and for some phases of care; adjusting for inflation alone would not result in accurate approximations of cost estimates (Table S9 in Appendix S2). Consistent with the previous analysis, surgery costs decreased overall but increased in terms of patient liability, and costs of nonsurgery treatments were lower for older patients. Our terminal phase costs for patients who died of lung cancer appear lower than in the previous study, which may be explained by our six‐month terminal phase compared with their one‐month terminal phase. Cost estimates using a one‐month terminal phase can be found in Appendix S2 (Table S10) and are comparable to previous estimates using the one‐month terminal phase.^10^ Other estimates of lung cancer treatment costs have used 12‐month initial and terminal phases,^9^, ^10^, ^23^ but because our data showed evidence of noticeably higher costs in the six months prior to death, we believe a six‐month terminal phase is the most accurate way to allocate costs for lung cancer patients.

Cancer‐attributable costs determined by matching cancer patients to noncancer controls have also been published.^9^ The percent of total Medicare spending attributed to lung cancer treatment has been shown to be the same using a self‐control or matching method.^24^ Our self‐control method enabled us to control for the prior presence of comorbid conditions and health‐related behaviors that may be correlated with healthcare costs, but are not included in SEER‐Medicare data, such as smoking status.^14^

Our granular, phase‐specific cost estimates will allow cost‐effectiveness analyses (CEA) to more accurately assess the economic value of lung cancer prevention, screening, and treatment options.^5^, ^6^, ^7^ Many analyses use either incomplete or outdated cost estimates. Several recent CEA studies comparing lung cancer treatment options used the outdated lung cancer treatment costs estimated from SEER‐Medicare data no later than 2003.^9^, ^10^ Another study, which estimated the incremental cost‐effectiveness ratio of screening with low‐dose CT, did not include any costs after initial treatment.^8^ Our cost estimates will enable CEA studies to be up to date and comprehensive with respect to multiple distinct phases of cancer care.

This analysis is subject to certain limitations. First, we were unable to estimate costs for immunotherapy drugs, which have shown promising gains in survival for some patients, because approvals for lung cancer treatment came after the end of our study period.^25^ Second, the SEER‐Medicare data used in our analysis were limited to patients over age 65 who were not simultaneously enrolled in managed care and we were unable to determine the proportion of patient‐liability costs paid out of pocket vs the portion paid by purchased Medigap coverage. SEER also only collects data from cancer registries in certain geographic areas, meaning our results may not be completely generalizable to the US population. Finally, it is possible that some costs may be misclassified according to our phase of care definitions. For example, a patient who died in early 2014 may have incurred costs that were misallocated to the initial or continuing phase because we did not have claims data later than 31 December 2013.

In this analysis, we assigned lung cancer treatment costs to phases of care by age, stage at diagnosis, histology, and treatment strategy. Baseline healthcare costs prior to lung cancer diagnosis rose, while costs during the final six months of life fell. Costs during the initial and continuing phases varied widely depending on treatment strategy. The cost of best supportive care remained stable for most groups. These cost estimates will be a crucial component of updated, comprehensive cost‐effectiveness analyses, which are essential to our understanding of lung cancer control interventions.

## CONFLICT OF INTEREST

The authors declare that there is no conflict of interest.
