# Supplementary material for: Lung cancer costs by treatment strategy and phase of care among patients enrolled in Medicare
Source: Cancer Med. 2018 Dec 21;8(1):94–103. doi: 10.1002/cam4.1896 (PMC6346221; doi:10.1002/cam4.1896)
Supplement: Supplementary file 1 [file CAM4-8-94-s001.pdf]

A: Supplementary Methods

Appendix

Sheehan et al. 2018

Contents

Page 3 – **Treatment Strategies:** Defining chemotherapy, radiation, and surgery

Page 4 – **Linear Regression Models:** Description of model parameters

Treatment Strategies

Defining chemotherapy, radiation, and surgery

Patients were defined as having received **chemotherapy** if they had inpatient, outpatient, physician, hospice, home health, or durable medical equipment claims with any of the following codes: International Classification of Diseases, 9th Revision, Clinical Modification (ICD-9-CM) procedure code 99.25 or ICD-9-CM diagnosis codes V58.1 or V58.11; revenue center codes 0331, 0332, or 0335; Healthcare Common Procedures Coding System (HCPCS) codes C1166, C1167, C1178, C9110, C9205, C9207, C9213-C9216, C9411, C9414-C9419, C9420-C9438, G0355, G0356, G0359-G0362, J7150, J8500-J8799, J8999-J9999, Q0083-Q0085, S9325-S9329, S9330-S9379, or S9494-S9497; or Current Procedure Terminology (CPT) codes 96400-96546.

Patients were defined as having received **radiation** therapy if they had inpatient, outpatient, physician, hospice, home health, or durable medical equipment claims with any of the following codes: ICD-9-CM procedure codes 92.2x or ICD-9-CM diagnosis code V58.0; revenue center codes 0330, 0333, or 0339; CPT codes 77000-77999 or 79000-79999; or HCPCS code S8049.

Patients were defined as having received **surgery** if they had wedge resection, pneumonectomy, or lobectomy. Patients were identified using the following ICD-9-CM procedure and CPT codes: ICD-9-CM codes 32.2x, 32.3x, 32.4x, 32.5x, 32.6x, or 32.9x; or CPT codes 32440, 32442, 32445, 32480, 32482, 32484, 32486, 32500, 32505, 32506, 32507, or 32520. Additional ICD-9-CM procedure codes 32.01, 32.09, 32.1x, 40.11, and 40.19 indicated that a patient had non-major surgery.

Linear regression models

Description of model parameters

For the initial and continuing phases, linear regression models were fit for each treatment strategy used for at least 10% of that stage/histology group. For example, in Extensive Stage SCLC patients, very few (<1%) patients were treated with surgery, so we did not calculate costs for surgery in these patients. The exception to this is best supportive care, which modeled for all groups. All cost outputs from these models are monthly costs.

The “age” variable in each model represents a patient’s age during that phase. For staging, surgery, and initial phase models, we used age of diagnosis as our age variable, because these phases occur within 7 months of diagnosis. A patient’s continuing phase may possibly span many years, so it would be inappropriate to base continuing phase costs on the age of diagnosis. We analyzed the continuing phase as age-based sub-phases, but for the purposes of these models, one should plug in a single numeric age into the model to calculate monthly cost. In the terminal phase models, age of death was used as the age variable, because the terminal phase occurs within 6 months of death.

The “year” variable in each model represents calendar year. It is important to note that the year variable has been scaled – for the year 2000, one should plug in “0”, for the year 2001, one should plug in “1”, and so on. For staging, surgery, and initial phase models, we used year of diagnosis as our year variable, because these phases occur within 7 months of diagnosis. Although a patient’s continuing phase may span many years, the year variable in the continuing phase models represents year during the age-based sub-phase. In the terminal phase models, year represents year of death.
