# Supplementary material for: Lung cancer costs by treatment strategy and phase of care among patients enrolled in Medicare
Source: Cancer Med. 2018 Dec 21;8(1):94–103. doi: 10.1002/cam4.1896 (PMC6346221; doi:10.1002/cam4.1896)
Supplement: Supplementary file 2 [file CAM4-8-94-s002.pdf]

B: Supplementary Results

Appendix

Sheehan et al. 2018

Contents

Page 3 – **Supplementary Figure B.1:** Average monthly costs 15 months prediagnosis to 6 months postdiagnosis

Page 4 – **Supplementary Figure B.2:** Support for the six-month terminal phase: Total monthly costs following lung cancer diagnosis, by survival time

Page 5 – **Supplementary Figure B.3:** Change in baseline healthcare costs during the prediagnosis phase

Page 6 – **Supplementary Table B.1:** Results of a sensitivity analysis to determine the effect of increasing baseline healthcare costs

Page 7 – **Supplementary Table B.2:** Number and percent of patients in each treatment group – Initial phase

Page 8 – **Supplementary Table B.3:** Number and percent of patients in each treatment group – Continuing phase

Page 9 – **Supplementary Table B.4:** Monthly prediagnosis phase healthcare costs by age and year and significant predictors

Page 10 – **Supplementary Table B.5:** Significant predictors of cost during the staging phase and costs for a patient aged 70 in 2017 by histology, stage at diagnosis, and treatment strategy

Page 12 – **Supplementary Table B.6:** Monthly terminal phase costs by age and year and significant predictors by cause of death

Page 13 – **Supplementary Table B.7:** Monthly prescription drug costs for an average patient age 70 in 2017

Page 14 – **Supplementary Table B.8:** Average monthly costs of erlotinib, by phase, 2007-2013

Page 15 – **Supplementary Table B.9:** Why not just inflate previous SEER-Medicare cost estimates?

Page 16 – **Supplementary Table B.10:** Cost estimates using a one-month terminal phase, for a patient who died at age 70 in 2017

Supplementary Figure B.1

Average monthly costs 15 months prediagnosis to 6 months postdiagnosis

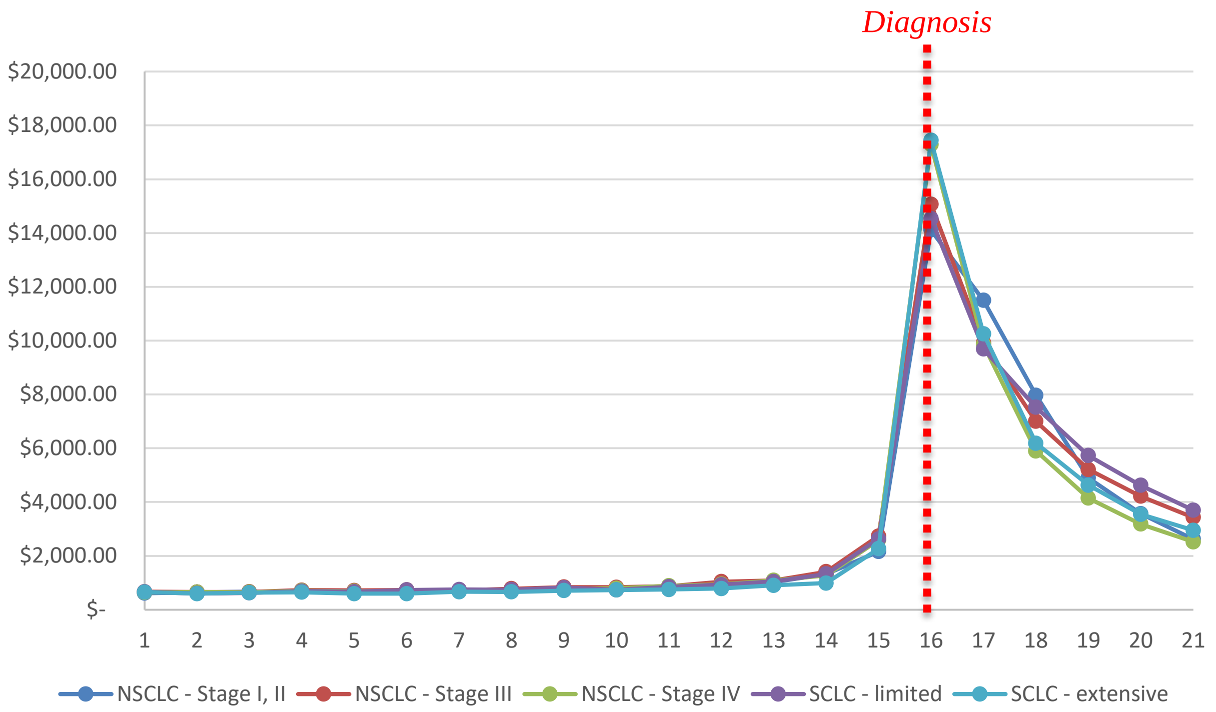

Supplementary Figure 1. This figure shows total monthly healthcare costs from 15 months prior to lung cancer diagnosis through 6 months post-diagnosis, where month 16 represents the month beginning on the date of diagnosis.

Supplementary Figure B.2

Support for the six-month terminal phase: Total monthly costs following lung cancer diagnosis, by survival time

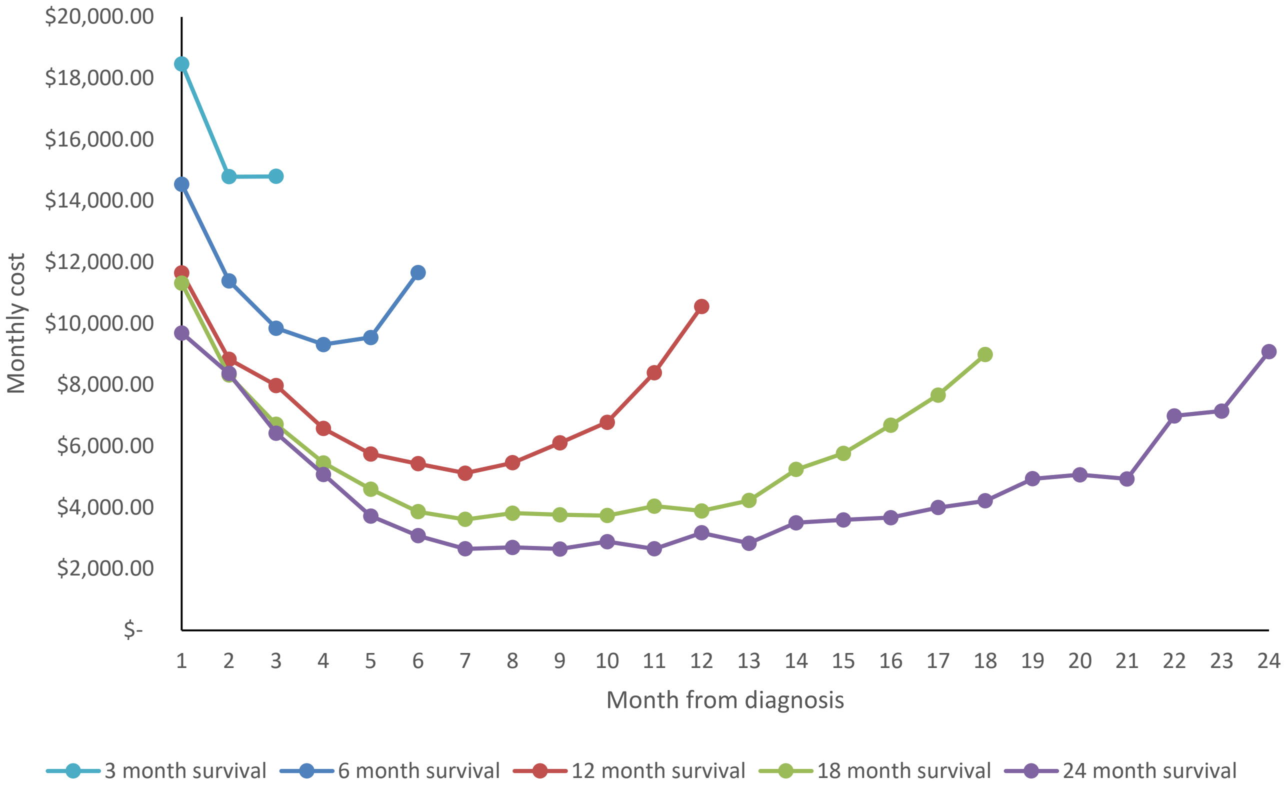

Supplementary Figure 2. Costs are high during the month of lung cancer diagnosis and/or surgery. Costs then begin to fall during the months categorized as the initial and continuing phases, and rise again prior to death. For patients who live at least one year, costs begin to rise again about 5-6 months prior to death. Our decision to use a six-month terminal phase is also supported by our finding that costs during the six months prior to death are, on average, 2.9 times as high as costs in the period 7-12 months before death. This figure supports our decision to have a six-month terminal phase.

Supplementary Figure B.3

Change in baseline healthcare costs during the prediagnosis phase

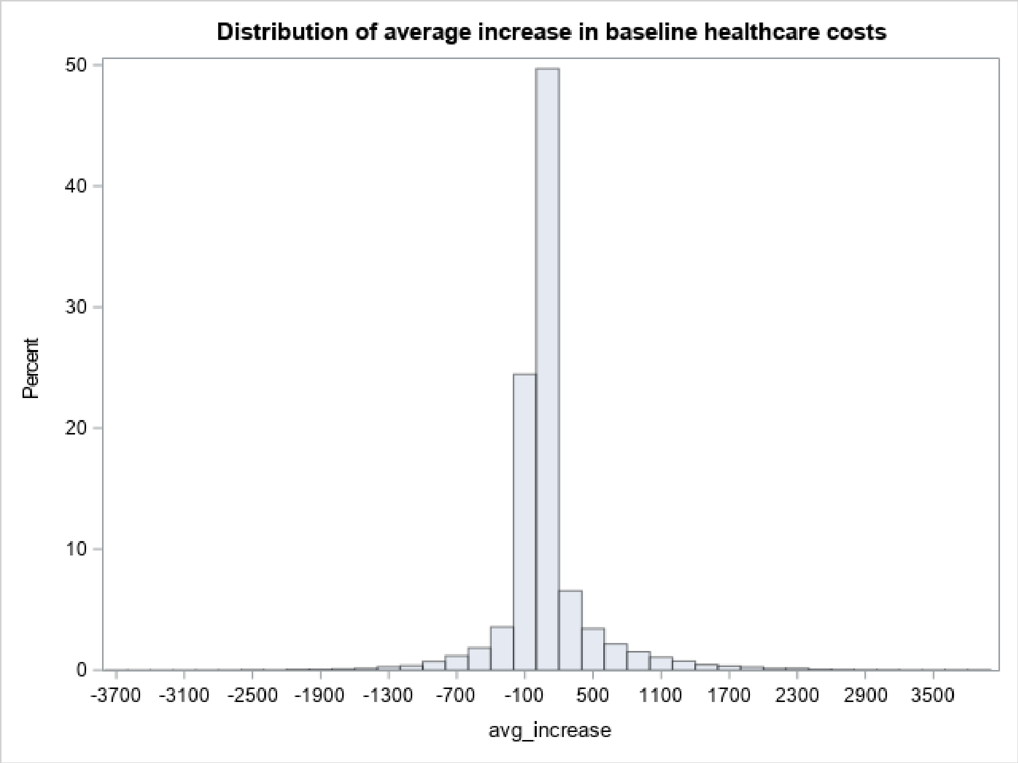

Supplementary Figure 3. We performed a sensitivity analysis to determine the effect that increasing baseline healthcare costs have on the cancer-attributable costs calculated in our analysis. This figure shows that the distribution of change in baseline healthcare costs was relatively normally distributed around 0, meaning that a large proportion of patients happened to see a decrease in healthcare costs during the five years prior to lung cancer diagnosis. Results of the sensitivity analysis can be found in Supplementary Table B.1.

Supplementary Table B.1

Results of a sensitivity analysis to determine the effect of increasing baseline healthcare costs

	Original	Accounting for rising baseline healthcare costs
Initial phase monthly cancer attributable total cost	$2,132	$2,064
Terminal phase monthly cancer attributable total cost	$9,877	$9,642

Supplementary Table 1. We calculated monthly healthcare costs for the five years prior to lung cancer diagnosis among the 27,208 early-stage lung cancer patients who had been enrolled in Medicare for that entire time-period. We then determined the average annual change in prediagnosis healthcare costs, and, assuming a linear increase in baseline healthcare costs, removed the annual average from the cancer-attributable healthcare costs post-diagnosis each year. Average monthly cancer-attributable costs during the initial phase decreased from $2,132 to $2,064, while average costs during the terminal phase decreased from $9,877 to $9,642. The relatively small change in cancer-attributable costs (3.2% for initial phase and 2.4% for terminal phase) among these early-stage patients has assured us that we are not significantly over-estimating patients' cancer-attributable costs.

We performed this sensitivity analysis among patients diagnosed with stage I or II lung cancer. Average post-diagnosis survival times for stages III and IV patients are 13.5 and 7.3 months, respectively, meaning that late-stage patients do not survive long enough for their baseline healthcare costs to increase much beyond what we originally used to represent baseline healthcare costs. Early-stage patients, on the other hand, often survive for years following their lung cancer diagnosis. Performing the sensitivity analysis among this group of patients enables us to determine the effect of rising baseline healthcare costs among patients with the highest average survival times during which baseline costs often rise due to comorbid conditions. As seen in Supplementary Figure B.3, many patients' baseline healthcare costs decreased during the prediagnosis period. For a patient who lives multiple years following lung cancer diagnosis, we do not believe it would be accurate to continue to linearly extrapolate decreasing healthcare costs as that patient ages. Thus, we included this analysis as a sensitivity analysis in the Appendix, rather than in the methods for how we report cancer-attributable costs in the main manuscript.

Supplementary Table B.2

Number and percent of patients in each treatment group – Initial phase

**INITIAL PHASE**	**n**	**%**
**NSCLC**	26,959	
**Stage 1-2**		
Best supportive care	3,913	14.51
Surgery	13,872	51.46
Radiation	3,453	12.81
Chemotherapy	416	1.54
Chemo + radiation	1,400	5.19
Chemo + surgery	1,233	4.57
Radiation + surgery	2,045	7.59
Chemo + radiation + surgery	627	2.33
**Stage 3**	16,282	
Best supportive care	3,335	20.48
Radiation	2,973	18.26
Chemotherapy	1,902	11.68
Chemo + radiation	4,509	27.69
Surgery	1,577	9.69
Radiation + surgery	648	3.98
Chemo + surgery	600	3.69
Chemo + radiation + surgery	738	4.53
**Stage 4**	13,583	
Best supportive care	1,830	13.47
Radiation	2,349	17.29
Chemotherapy	2,050	15.09
Chemo + radiation	6,283	46.26
Surgery	136	1.0
Radiation + surgery	255	1.88
Chemo + surgery	175	1.29
Chemo + radiation + surgery	505	3.72

continued	**n**	**%**
**SCLC**		
**Limited Stage**	10,331	
Best supportive care	1,581	15.3
Chemotherapy	1,523	14.74
Radiation	1,871	18.11
Chemo + radiation	4,057	39.27
Surgery	750	7.26
Chemo + surgery	223	2.16
Radiation + surgery	144	1.39
Chemo + radiation + surgery	182	1.76
**Extensive Stage***	7,591	
Best supportive care	445	5.86
Chemotherapy	1,364	17.97
Radiation	736	9.7
Chemo + radiation	4,917	64.77
Chemo + radiation + surgery	84	1.11

*Additional treatment combinations each made up less than 1% of treatment strategies for SCLC extensive stage patients.

Supplementary Table B.3

Number and percent of patients in each treatment group – Continuing phase

CONTINUING PHASE	n	%
**NSCLC**		
**Stage 1-2**	23,712	
Best supportive care	3,199	13.49
Surgery	13,197	55.66
Radiation	2,586	10.91
Chemotherapy	287	1.21
Chemo + radiation	955	4.03
Surgery + Radiation	1,839	7.76
Surgery + Chemo	1,124	4.74
Surgery + chemo + radiation	525	2.21
**Stage 3**	10,960	
Best supportive care	2,255	20.57
Chemotherapy	1,184	10.8
Chemo + radiation	2,738	24.98
Radiation	1,813	16.54
Surgery	1,395	12.73
Surgery + chemo	507	4.63
Surgery + radiation	510	4.65
Surgery + chemo + radiation	558	5.09
**Stage 4**	7,443	
Best supportive care	893	12
Chemotherapy	1,124	15.1
Chemo + radiation	3,512	47.19
Radiation	1,091	14.66
Surgery	108	1.45
Surgery + chemo	128	1.72
Surgery + radiation	193	2.59
Surgery + chemo + radiation	394	5.29

continued	n	%
**SCLC**		
**Limited Stage**	6,928	
Best supportive care	1,154	16.66
Chemotherapy	868	12.53
Radiation	1,280	18.48
Chemo + radiation	2,508	36.2
Surgery	675	9.74
Surgery + chemo	188	2.71
Surgery + radiation	108	1.56
Surgery + chemo + radiation	147	2.12
**Extensive Stage***	3,476	
Best supportive care	198	5.7
Chemotherapy	561	16.14
Radiation	315	9.06
Chemo + radiation	2,316	66.63
Chemo + radiation + surgery	56	1.61

*Additional treatment combinations each made up less than 1% of treatment strategies for SCLC extensive stage patients.

Supplementary Table B.4

Monthly prediagnosis phase healthcare costs by age and year and significant predictors*

				Predictors	
	2000	2010	2017	Year	Age
**Total cost**				+	+
65	$516	$688	$809		
70	$568	$740	$861		
75	$620	$792	$913		
80	$672	$844	$965		
**Patient liability cost**				+	+
65	$60	$70	$77		
70	$67	$77	$84		
75	$75	$85	$92		
80	$82	$92	$99		

** A positive (+) symbol indicates that the covariate in the regression model has a parameter estimate greater than 0, while a negative (-) symbol indicates that the parameter estimate is less than 0. Coefficients for the fitted regressions are presented in Appendix C: Regression Parameters.*

Supplementary Table B.5

Significant predictors of cost during the staging phase and costs for a patient aged 70 in 2017 by histology, stage at diagnosis, and treatment strategy*

						Predictors	
	N (%)	Total cost	95% CI	Patient liability	95% CI	Year	Age
NSCLC							
**Stages I and II**	9,577						
Best supportive care	4,064 (42.4)	$6,670	$6,014 to $7,327	$677	$610 to $744		+
Radiation	3,610 (37.7)	$9,739	$8,957 to $10,522	$872	$712 to $1,033		–
Chemotherapy + Radiation	1,467 (15.3)	$8,576	$7,885 to $9,267	$1,166	$1,111 to $1,220		–
**Stage III**	13,812						
Best supportive care	3,608 (26.1)	$9,560	$8,765 to $10,354	$905	$820 to $989		+
Radiation	3,273 (23.7)	$10,636	$10,104 to $11,168	$1,264	$1,217 to $1,312		
Chemotherapy	2,008 (14.5)	$10,476	$9,897 to $11,055	$873	$720 to $1,025		
Chemotherapy + Radiation	4,923 (35.6)	$9,722	$9,272 to $10,171	$944	$832 to $1,057		–
**Stage IV**	14,248						
Best supportive care	2,167 (15.2)	$12,961	$11,972 to $13,950	$1,271	$1,176 to $1,366		
Radiation †	2,801 (19.7)	$13,608	$10,399 to $16,818	$960	$676 to $1,244	–	–
Chemotherapy	2,300 (16.1)	$11,311	$10,451 to $12,171	$876	$688 to $1,063		–
Chemotherapy + Radiation	6,980 (49.0)	$9,627	$8,454 to $10,800	$1,033	$938 to $1,128	–	–

See following page for SCLC results and footnotes.

Continued: Significant predictors of cost during the staging phase and costs for a patient aged 70 in 2017 by histology, stage at diagnosis, and treatment strategy

						Predictors	
	N (%)	Total cost	95% CI	Patient liability	95% CI	Year	Age
SCLC							
**Limited Stage**	9,645						
Best supportive care	1,669 (17.3)	$7,397	$6,067 to $8,728	$756	$648 to $864		+
Radiation	2,028 (21.0)	$8,808	$8,217 to $9,399	$661	$379 to $944		
Chemotherapy	1,623 (16.8)	$11,346	$10,543 to $12,149	$852	$600 to $1,105		
Chemotherapy + Radiation	4,325 (44.8)	$11,057	$10,524 to $11,589	$1,209	$1,070 to $1,348		–
**Extensive Stage**	8,411						
Best supportive care	535 (6.4)	$10,697	$9,377 to $12,017	$1,003	$877 to $1,129		
Radiation	895 (10.6)	$12,631	$11,443 to $13,819	$1,383	$1,267 to $1,499		
Chemotherapy	1,574 (18.7)	$10,431	$9,778 to $11,083	$820	$549 to $1,092		
Chemotherapy + Radiation	5,407 (64.3)	$12,852	$12,360 to $13,345	$1,259	$1,143 to $1,375		–

* The directions of significant predictors are shown for linear regression models of total costs. A positive (+) symbol indicates that the covariate in the regression model has a parameter estimate greater than 0, while a negative (-) symbol indicates that the parameter estimate is less than 0. With the exception of best supportive care costs, treatment strategy costs are not shown if less than 10% of patients within a stage/histology group received that treatment. Coefficients for the fitted regressions are presented in the Appendix.
† The interaction term between year and age was significant in the model for Stage IV NSCLC patients who received radiation. The parameter estimate of the interaction term was positive, so costs decreased with age but increased over time.

Supplementary Table B.6

Monthly terminal phase costs by age and year and significant predictors by cause of death*

					Predictors		
		2000	2010	2017	Year	Age	Year* age
**Lung cancer death**							
Total cost †					+	–	–
	65	$14,795	$14,506	$14,305			
	70	$14,053	$13,426	$12,987			
	75	$13,311	$12,345	$11,669			
	80	$12,570	$11,265	$10,351			
Patient liability cost ‡						–	–
	65	$1,439	$1,350	$1,288			
	70	$1,347	$1,238	$1,161			
	75	$1,256	$1,126	$1,035			
	80	$1,165	$1,014	$909			
**Non-lung cancer death**							
Total cost					–	–	
	65	$20,351	$15,247	$11,673			
	70	$18,944	$13,840	$10,266			
	75	$17,537	$12,433	$8,859			
	80	$16,131	$11,026	$7,452			
Patient liability cost ‡						–	–
	65	$1,496	$1,232	$1,048			
	70	$1,422	$1,126	$919			
	75	$1,349	$1,020	$789			
	80	$1,276	$914	$660			

* A positive (+) symbol indicates that the covariate in the regression model has a parameter estimate greater than 0,
while a negative (-) symbol indicates that the parameter estimate is less than 0. Coefficients for the fitted regressions are presented in the Appendix.
† The interaction term between year and age was significant and negative in the model for total lung cancer death cost. Costs decreased over time, even though the coefficient for year was positive in the model.
‡ The interaction term between year and age was significant in the models for lung cancer death and non-lung cancer death patient liability cost. Year was kept in the models although it was not significant (p=0.07 for lung cancer death, p=0.39 for non-lung cancer death) to ensure the model's terms were hierarchically well-formulated.

Supplementary Table B.7

Monthly prescription drug costs for an average patient age 70 in 2017

	Estimate	95% CI
**Prediagnosis phase**	$ 651	$636 to $666
**Surgery phase**	$ 604	$562 to $646
**Staging phase**	$ 992	$928 to $1,057
**Initial phase, cancer-attributable cost**		
Best supportive care	$ 845	$646 to $1,045
Surgery	$ 221	$181 to $261
Chemotherapy	$ 875	$641 to $1,108
Radiation	$ 487	$361 to $613
Chemotherapy + radiation	$ 675	$576 to $774
**Continuing phase, cancer-attributable cost**		
Best supportive care	$ 120	$61 to $178
Surgery	$ 28	$5 to $51
Chemotherapy	$ 279	$188 to $370
Radiation	$ 26	($25) to $77
Chemotherapy + radiation	$ 198	$156 to $240
**Terminal phase**	$ 1050	$954 to $1,146

Supplementary Table B.8

Average monthly costs of erlotinib, by phase, 2007-2013

	n	Total cost	95% CI	Patient liability cost	95% CI
Initial phase	2596	$671	$610 to $732	$98	$85 to $112
Continuing phase	2114	$1484	$1405 to $1564	$135	$123 to $148
Terminal phase	2723	$1844	$1777 to $1912	$252	$236 to $268

Supplementary Table B.9

Why not just inflate previous SEER-Medicare cost estimates?

	Previous cost manuscript, 2000 costs in 2006 USD	Old costs inflated 3% per year to 2017 USD	Cost update, 2000 costs in 2017 USD
Age 65	$ 554	$ 722	$ 516
Age 70	$ 619	$ 807	$ 568
Age 75	$ 683	$ 892	$ 620
Age 80	$ 748	$ 976	$ 672

Supplementary Table B.10

Cost estimates using a one-month terminal phase, for a patient who died at age 70 in 2017

	**Six-month terminal phase**	**One-month terminal phase**
Lung cancer death	$12,987	$21,603
Other cause of death	$10,266	$23,179
