# Supplementary material for: Lung cancer costs by treatment strategy and phase of care among patients enrolled in Medicare
Source: Cancer Med. 2018 Dec 21;8(1):94–103. doi: 10.1002/cam4.1896 (PMC6346221; doi:10.1002/cam4.1896)
Supplement: Supplementary file 3 [file CAM4-8-94-s003.pdf]

C: Regression parameters

Appendix

Sheehan et al. 2018

Contents

Page 3 – **Parameter estimates of linear regression models**

Page 3 – Prediagnosis phase

Page 3 – Surgery phase

Page 4 – Staging phase

Page 10 – Initial phase

Page 17 – Continuing phase

Page 24 – Terminal phase

Page 25 – **Parameter estimates of linear regression models: Medicare Part D**

Page 25 – Prediagnosis phase: Part D

Page 25 – Surgery phase: Part D

Page 26 – Staging phase: Part D

Page 27 – Initial phase: Part D

Page 28 – Continuing phase: Part D

Page 29 – Terminal phase: Part D

Linear regression models – continued

PREDIAGNOSIS PHASE

Parameter	Parameter Estimate	Standard error	P-value
**Prediagnosis phase – total cost model**			
Intercept	-161.88817	56.72401	0.0043
Year	17.24651	1.4078	<.0001
Age	10.42334	0.73743	<.0001
**Prediagnosis phase – patient liability cost model**			
Intercept	-34.67437	5.5127	<.0001
Year	1.00019	0.13682	<.0001
Age	1.45677	0.07167	<.0001

SURGERY PHASE

Parameter	Parameter Estimate	Standard error	P-value
**Surgery phase – total cost model**			
Intercept	34471	612.31495	<.0001
Year	-257.34871	94.97436	0.0067
**Surgery phase – patient liability cost model**			
Intercept	1363.60608	30.89565	<.0001
Year	22.05216	4.79213	<.0001
**Operative death – total cost model**			
Intercept	149010	8975.8405	<.0001
Year	3614.24227	1461.0392	0.0135

Linear regression models – continued

STAGING PHASE: Stage I, II NSCLC

Parameter	Parameter Estimate	Standard error	P-value
**Best supportive care – total cost model**			
Intercept	-4052.62999	2472.0802	0.1012
Age	153.18426	31.60036	<.0001
**Best supportive care – patient liability model**			
Intercept	-842.887	253.2983	0.0009
Age	21.71117	3.23789	<.0001
**Radiation – total cost model**			
Intercept	16900	2910.8215	<.0001
Age	-102.30212	36.87958	0.0056
**Radiation – patient liability cost model**			
Intercept	1304.694	51.57009	<.0001
Year	-25.4311	7.1608	0.0004
**Chemotherapy + radiation – total cost model**			
Intercept	17000	3478.5806	<.0001
Age	-120.34813	46.21619	0.0093
**Chemotherapy + radiation – patient liability cost**			
No significant predictors. SD = 1069			

Linear regression models – continued

STAGING PHASE: Stage III NSCLC

Parameter	Parameter Estimate	Standard error	P-value
**Best supportive care – total cost model**			
Intercept	-4022.71436	2906.0321	0.1664
Age	194.03953	36.95381	<.0001
**Best supportive care – patient liability cost model**			
Intercept	-803.621	309.8075	0.0095
Age	24.40427	3.93959	<.0001
**Radiation – total cost**			
No significant predictors. SD = 15540			
**Radiation – patient liability cost**			
No significant predictors. SD = 1390			
**Chemotherapy – total cost**			
No significant predictors. SD = 13227			
**Chemotherapy – patient liability cost**			
Intercept	1286.51	37.09647	<.0001
Year	-24.3272	6.17	<.0001
**Chemotherapy + radiation – total cost model**			
Intercept	17752	2552.7498	<.0001
Age	-114.7186	34.35076	0.0008
**Chemotherapy + radiation – patient liability cost model**			
Intercept	1456.53	32.81529	<.0001
Year	-30.1217	4.8651	<.0001

Linear regression models – continued

STAGING PHASE: Stage IV NSCLC

Parameter	Parameter Estimate	Standard error	P-value
**Best supportive care – total cost model**			
No significant predictors. SD = 23473			
**Best supportive care – patient liability cost**			
No significant predictors. SD = 2258			
**Radiation – total cost model**			
Intercept	42028	7689.6185	<.0001
Age	-384.63833	100.757	0.0001
Year	-2220.44811	1084.5337	0.0407
Age*year interaction	30.46413	14.05493	0.0303
**Radiation – patient liability cost model**			
Intercept	3592.16	680.481	<.0001
Age	-27.3405	8.91634	0.0022
Year	-310.945	95.97415	0.0012
Age*year interaction	3.83828	1.24377	0.002
**Chemotherapy – total cost model**			
Intercept	28996	4332.0333	<.0001
Age	-252.64527	57.465	<.0001
**Chemotherapy – patient liability cost model**			
Intercept	2330.385	320.6435	<.0001
Age	-14.2002	4.26111	0.0009
Year	-27.1019	7.32543	0.0002
**Chemotherapy + radiation – total cost model**			
Intercept	23602	2468.1868	<.0001
Age	-163.12211	33.37285	<.0001
Year	-150.36202	51.05097	0.0032

Linear regression models – continued

Continued – STAGING PHASE: Stage IV NSCLC

Parameter	Parameter Estimate	Standard error	P-value
**Chemotherapy + radiation – patient liability cost model**			
Intercept	2348.931	199.7462	<.0001
Age	-12.6817	2.70081	<.0001
Year	-25.2072	4.13147	<.0001

Linear regression models – continued

STAGING PHASE: Limited Stage SCLC

Parameter	Parameter Estimate	Standard error	P-value
**Best supportive care – total cost model**			
Intercept	-6058.60985	5475.7994	0.2687
Age	192.22943	70.82557	0.0067
**Best supportive care – patient liability cost model**			
Intercept	-995.754	445.6626	0.0256
Age	25.02324	5.76433	<.0001
**Radiation – total cost model**			
No significant predictors. SD=13574			
**Radiation – patient liability cost model**			
Intercept	1420.079	74.94313	<.0001
Year	-44.6302	11.98831	0.0002
**Chemotherapy – total cost model**			
No significant predictors. SD=16492			
**Chemotherapy – patient liability cost model**			
Intercept	1355.077	50.27994	<.0001
Year	-29.5661	9.8051	0.0026
**Chemotherapy + radiation – total cost model**			
Intercept	20328	3129.8432	<.0001
Age	-132.44477	42.31167	0.0018
**Chemotherapy + radiation – patient liability cost model**			
Intercept	1466.358	37.45487	<.0001
Year	-15.1607	5.92479	0.0105

Linear regression models – continued

STAGING PHASE: Extensive Stage SCLC

Parameter	Parameter Estimate	Standard error	P-value
**Best supportive care – total cost**			
No significant predictors. SD=15542			
**Best supportive care – patient liability cost**			
No significant predictors. SD=1482			
**Radiation – total cost**			
No significant predictors. SD=18111			
**Radiation – patient liability cost**			
No significant predictors. SD=1770			
**Chemotherapy – total cost**			
No significant predictors			
**Chemotherapy – patient liability cost model**			
Intercept	1368.97	59.98028	<.0001
Year	-32.2634	10.92992	0.0032
**Chemotherapy + radiation – total cost model**			
Intercept	26876	3038.7687	<.0001
Age	-200.34107	41.32708	<.0001
**Chemotherapy + radiation – patient liability cost model**			
Intercept	1496.123	34.27684	<.0001
Year	-13.9579	5.12249	0.0065

Linear regression models – continued

INITIAL PHASE: Stage I, II NSCLC

Paramet er	Parameter Estimate	Standard error	P-value
**Best supportive care – total cost model**			
Intercept	2239.07028	139.55441	<.0001
Year	53.34942	22.64084	0.0185
**Surgery – total cost model**			
Intercept	1298.6318	57.44912	<.0001
Year	22.17895	9.30905	0.0172
**Radiation – total cost model**			
Intercept	7311.77448	1044.1386	<.0001
Age	-42.13772	13.22884	0.0015
Year	91.71285	23.32667	<.0001

Parame ter	Parameter Estimate	Standard error	P-value
**Best supportive care – cancer attributable cost**			
No significant predictors. SD =4968			
**Surgery – cancer attributable cost model**			
Intercept	-14.63398	458.69827	0.9745
Age	12.0406	6.10808	0.0487
**Radiation – cancer attributable cost model**			
Intercept	3183.3687	172.75736	<.0001
Year	62.29857	23.88229	0.0091

Paramet er	Parameter Estimate	Standard error	P-value
**Best supportive care – patient liability cost**			
No significant predictors. SD=479			
**Surgery – patient liability cost**			
No significant predictors. SD=453			
**Radiation – patient liability cost model**			
Intercept	1177.17289	145.3694	<.0001
Year	-9.32439	3.24764	0.0041
Age	-4.04327	1.84178	0.0282

Linear regression models – continued

INITIAL PHASE: Stage III NSCLC

Paramet er	Parameter Estimate	Standard error	P-value
**Best supportive care – total cost**			
No significant predictors. SD=4434			
**Chemotherapy + radiation – total cost model**			
Intercept	12739	1278.9054	<.0001
Age	-68.47132	17.22935	<.0001
**Surgery – total cost model**			
Intercept	1565.27897	219.51051	<.0001
Year	102.7439	37.93277	0.0068
**Radiation – total cost model**			
Intercept	8808.54185	1372.6684	<.0001
Age	-49.19456	17.82718	0.0058
Year	104.76321	32.41248	0.0012
**Chemotherapy – total cost model**			
Intercept	10255	1701.165	<.0001
Age	-56.66025	22.49716	0.0119
Year	140.58843	36.19448	0.0001

Paramet er	Parameter Estimate	Standard error	P-value
**Best supportive care – cancer attributable cost**			
No significant predictors. SD=4510			
**Chemotherapy + radiation – cancer attributable cost model**			
Intercept	12140	1303.6911	<.0001
Age	-66.3364	17.56326	0.0002
**Surgery – cancer attributable cost model**			
Intercept	1173.4976	220.90966	<.0001
Year	87.62112	38.17455	0.0219
**Radiation – cancer attributable cost model**			
Intercept	7675.6687	1407.56	<.0001
Age	-42.66361	18.28032	0.0197
Year	79.2859	33.23636	0.0171
**Chemotherapy – cancer attributable cost model**			
Intercept	9823.1724	1731.0318	<.0001
Age	-57.10611	22.89214	0.0127
Year	136.47376	36.82994	0.0002

Linear regression models – continued

Continued – INITIAL PHASE: Stage III NSCLC

Parameter	Parameter Estimate	Standard error	P-value
**Best supportive care – patient liability cost model**			
Intercept	311.60952	15.65874	<.0001
Year	-5.74915	2.58647	0.0263
**Chemotherapy + radiation – patient liability cost model**			
Intercept	1977.47776	177.6522	<.0001
Year	-19.775	3.53398	<.0001
Age	-7.03968	2.39354	0.0033
**Surgery – patient liability cost model**			
Intercept	176.24165	41.053	<.0001
Year	16.78235	7.09421	0.0181
**Radiation – patient liability cost model**			
Intercept	1493.26156	197.5366	<.0001
Year	-19.931	4.66438	<.0001
Age	-5.89452	2.56546	0.0217
**Chemotherapy – patient liability cost model**			
Intercept	1631.81243	235.4783	<.0001
Year	11.57264	5.01011	0.021
Age	-8.98038	3.1141	0.004

Linear regression models – continued

INITIAL PHASE: Stage IV NSCLC

Parameter	Parameter Estimate	Standard error	P-value
**Best supportive care – total cost model**			
Intercept	6361.6374	1522.3681	<.0001
Age	-35.00087	19.02618	0.066
**Radiation – total cost model**			
Intercept	13873	1796.9427	<.0001
Age	-109.67561	23.12079	<.0001
**Chemotherapy + radiation – total cost model**			
Intercept	11030	1154.8012	<.0001
Age	-65.83239	15.6061	<.0001
Year	136.33155	24.08269	<.0001
**Chemotherapy – total cost model**			
Intercept	5361.2644	240.44971	<.0001
Year	108.07336	39.8926	0.0068

Parameter	Parameter Estimate	Standard error	P-value
**Best supportive care – cancer attributable cost model**			
Intercept	5772.6898	1554.5199	0.0002
Age	-39.73328	19.428	0.041
**Radiation – cancer attributable cost model**			
Intercept	13068	1801.4773	<.0001
Age	-109.54642	23.17914	<.0001
**Chemotherapy + radiation – cancer attributable cost model**			
Intercept	11210	1161.4943	<.0001
Age	-73.06838	15.69655	<.0001
Year	128.94812	24.22227	<.0001
**Chemotherapy – cancer attributable cost model**			
Intercept	4842.4092	247.92291	<.0001
Year	116.63074	41.13246	0.0046

Linear regression models – continued

Continued – INITIAL PHASE: Stage IV NSCLC

Paramet er	Parameter Estimate	Standard error	P-value
**Best supportive care – patient liability cost**			
No significant predictors. SD =708			
**Radiation – patient liability cost model**			
Intercept	847.68797	38.8699	<.0001
Year	-18.73896	5.56347	0.0008
**Chemotherapy + radiation – patient liability cost model**			
Intercept	2036.01275	166.6246	<.0001
Age	-12.10445	2.25292	<.0001
**Chemotherapy – patient liability cost model**			
Intercept	1670.63711	247.662	<.0001
Age	-10.41769	3.28805	0.0016

Linear regression models – continued

INITIAL PHASE: Limited Stage SCLC

Parameter	Parameter Estimate	Standard error	P-value
**Best supportive care – total cost**			
No significant predictors. SD=4128			
**Chemotherapy – total cost**			
No significant predictors. SD=8388			
**Radiation – total cost**			
Intercept	9723.86535	1567.9495	<.0001
Age	-59.93773	20.29881	0.0032
**Chemotherapy + radiation – total cost model**			
Intercept	10278	1240.6262	<.0001
Age	-40.6714	16.75188	0.0152
Year	68.57056	27.04152	0.0113

Parameter	Parameter Estimate	Standard error	P-value
**Best supportive care – cancer attributable cost model**			
No significant predictors. SD=4214			
**Chemotherapy – cancer attributable cost**			
No significant predictors. SD=8409			
**Radiation – cancer attributable cost model**			
Intercept	9046.379	1665.0152	<.0001
Age	-62.35797	21.55543	0.0039
**Chemotherapy + radiation – cancer attributable cost model**			
Intercept	10435	1228.8451	<.0001
Age	-43.87048	16.63906	0.0084

Parameter	Parameter Estimate	Standard error	P-value
**Best supportive care – patient liability cost**			
No significant predictors. SD=540			
**Chemotherapy – patient liability cost**			
No significant predictors. SD=766			
**Radiation – patient liability cost model**			
Intercept	1481.46782	210.3441	<.0001
Age	-5.63685	2.7324	0.0393
Year	-29.75461	5.71717	<.0001
**Chemotherapy + radiation – patient liability cost model**			
Intercept	1414.90569	24.89743	<.0001
Year	-14.11301	3.93173	0.0003

Linear regression models – continued

INITIAL PHASE: Extensive Stage SCLC

Parameter	Parameter Estimate	Standard error	P-value
**Best supportive care – total cost**			
No significant predictors. SD=4559			
**Chemotherapy – total cost**			
No significant predictors. SD=5577			
**Chemotherapy + radiation – total cost model**			
Intercept	10236	1387.7313	<.0001
Age	-39.80772	18.88992	0.0351

Parameter	Parameter Estimate	Standard error	P-value
**Best supportive care – cancer attributable cost**			
No significant predictors. SD=4664			
**Chemotherapy – cancer attributable cost**			
No significant predictors. SD=5669			
**Chemotherapy + radiation – cancer attributable cost model**			
Intercept	9942.2633	1396.6546	<.0001
Age	-41.31837	19.01138	0.0298

Parameter	Parameter Estimate	Standard error	P-value
**Best supportive care – patient liability cost model**			
Intercept	-2116.23535	880.3347	0.0167
Age	32.82355	11.45453	0.0044
Year	324.67255	144.6434	0.0254
Age*year interaction	-4.35144	1.88016	0.0212
**Chemotherapy – patient liability cost model**			
Intercept	1065.67125	45.70393	<.0001
Year	-17.50649	8.36892	0.0367
**Chemotherapy + radiation – patient liability cost model**			
Intercept	1713.03673	185.8473	<.0001
Age	-6.84376	2.51997	0.0066
Year	-10.32031	3.95308	0.0091

Linear regression models – continued

CONTINUING PHASE: Stage I, II NSCLC

Parameter	Parameter Estimate	Standard error	P-value
**Best supportive care – total cost model**			
Intercept	1813.7369	109.10296	<.0001
Year	51.50683	14.92613	0.0006
**Surgery – total cost model**			
Intercept	1268.01941	39.21853	<.0001
Year	35.37497	5.06031	<.0001
**Radiation – total cost model**			
Intercept	5576.61908	754.71481	<.0001
Year	42.48555	16.1346	0.0085
Age	-45.62408	9.8029	<.0001

Parameter	Parameter Estimate	Standard error	P-value
**Best supportive care – cancer attributable cost model**			
Intercept	1223.4874	113.16126	<.0001
Year	31.75026	15.48133	0.0403
**Surgery – cancer attributable cost model**			
Intercept	814.33504	42.16681	<.0001
Year	31.11871	5.44073	<.0001
**Radiation – cancer attributable cost model**			
Intercept	3640.6578	785.71942	<.0001
Age	-28.11105	10.1961	0.0059

Parameter	Parameter Estimate	Standard error	P-value
**Best supportive care – patient liability cost model**			
Intercept	204.31403	12.0452	<.0001
Year	4.38181	1.64788	0.0079
**Surgery – patient liability cost model**			
Intercept	157.16846	4.91022	<.0001
Year	4.97638	0.63356	<.0001
**Radiation – patient liability cost model**			
Intercept	633.67088	82.73414	<.0001
Year	6.14428	1.76872	0.0005
Age	-5.2965	1.07462	<.0001

Linear regression models – continued

CONTINUING PHASE: Stage III NSCLC

Param eter	Parameter Estimate	Standard error	P-value
**Best supportive care – total cost model**			
Intercept	4803.26833	862.30367	<.0001
Year	86.7937	20.12103	<.0001
Age	-35.16769	11.38563	0.002
**Chemotherapy – total cost model**			
Intercept	11328	1595.6532	<.0001
Year	171.88066	33.94188	<.0001
Age	-113.36235	21.60077	<.0001
**Radiation – total cost model**			
Intercept	6667.00444	1102.4165	<.0001
Year	73.45774	24.79273	0.0031
Age	-57.24448	14.73385	0.0001
**Chemotherapy + radiation – total cost model**			
No significant predictors. SD=4055			
**Surgery – total cost model**			
Intercept	1451.37557	138.21613	<.0001
Year	77.05078	19.04548	<.0001

Parame ter	Parameter Estimate	Standard error	P-value
**Best supportive care – cancer attributable cost model**			
Intercept	4619.7225	910.58189	<.0001
Year	89.18294	21.24755	<.0001
Age	-41.04207	12.02308	0.0007
**Chemotherapy – cancer attributable cost model**			
Intercept	10131	1655.0483	<.0001
Year	164.35349	35.2053	<.0001
Age	-102.41618	22.40482	<.0001
**Radiation – cancer attributable cost model**			
Intercept	6109.7707	1134.2489	<.0001
Year	55.57757	25.50862	0.0295
Age	-56.97012	15.15929	0.0002
**Chemotherapy + radiation – cancer attributable cost model**			
No significant predictors. SD=4137			
**Surgery – cancer attributable cost model**			
Intercept	1149.9053	138.93642	<.0001
Year	59.82852	19.14474	0.0018

Linear regression models – continued

Continued – CONTINUING PHASE: Stage III NSCLC

Parameter	Parameter Estimate	Standard error	P-value
**Best supportive care – patient liability cost model**			
Intercept	817.50656	113.2859	<.0001
Year	11.33972	2.64342	<.0001
Age	-7.33845	1.4958	<.0001
**Chemotherapy – patient liability cost model**			
Intercept	1833.58161	232.5926	<.0001
Year	18.77798	4.94758	0.0002
Age	-17.94934	3.14867	<.0001
**Radiation – patient liability cost model**			
Intercept	954.76425	127.5967	<.0001
Age	-8.95092	1.70534	<.0001
Year	11.06002	2.86958	0.0001
**Chemotherapy + radiation – patient liability cost model**			
Intercept	884.78392	114.4295	<.0001
Age	-6.90615	1.5605	<.0001
**Surgery – patient liability cost model**			
Intercept	190.09341	15.87735	<.0001
Year	9.80532	2.18782	<.0001

Linear regression models – continued

CONTINUING PHASE: Stage IV NSCLC

Parameter	Parameter Estimate	Standard error	P-value
**Best supportive care – total cost model**			
Intercept	1703.25028	215.49493	<.0001
Year	75.27901	30.34411	0.0133
**Chemotherapy – total cost model**			
Intercept	7265.35748	1392.1704	<.0001
Year	118.79797	31.4403	0.0002
Age	-55.65882	18.91564	0.0033
**Chemotherapy + radiation – total cost model**			
Intercept	304.08443	2227.2017	0.8914
Year	1063.73674	274.08526	0.0001
Age	41.92552	30.79338	0.1734
Age*year interaction	-12.72415	3.76833	0.0007
**Radiation – total cost model**			
Intercept	7571.70278	1355.4688	<.0001
Age	-64.11512	17.79911	0.0003

Parameter	Parameter Estimate	Standard error	P-value
**Best supportive care – cancer attributable cost model**			
No significant predictors. SD=3534			
**Chemotherapy – cancer attributable cost model**			
Intercept	6975.6669	1503.1132	<.0001
Year	132.30799	33.9458	0.0001
Age	-58.8683	20.42304	0.004
**Chemotherapy + radiation – cancer attributable cost model**			
Intercept	938.09597	2251.8469	0.677
Year	993.26282	277.11815	0.0003
Age	28.19398	31.13413	0.3652
Age*year interaction	-11.8144	3.81003	0.0019
**Radiation – cancer attributable cost model**			
Intercept	6067.0519	1406.2242	<.0001
Age	-55.23009	18.46559	0.0028

Linear regression models – continued

Continued – CONTINUING PHASE: Stage IV NSCLC

Parameter	Parameter Estimate	Standard error	P-value
**Best supportive care – patient liability cost**			
No significant predictors. SD=423			
**Chemotherapy – patient liability cost**			
Intercept	1336.76785	206.8615	<.0001
Year	15.38007	4.67169	0.001
Age	-11.18087	2.81066	<.0001
**Chemotherapy + radiation – patient liability cost**			
Intercept	554.00704	360.148	0.1241
Year	129.74238	44.32075	0.0034
Age	0.10585	4.97942	0.983
Age*year interaction	-1.50974	0.60935	0.0133
**Radiation – patient liability cost**			
Intercept	881.52484	172.5596	<.0001
Age	-7.61986	2.26594	0.0008

Linear regression models – continued

CONTINUING PHASE: Limited Stage SCLC

Parameter	Parameter Estimate	Standard error	P-value
**Best supportive care – total cost**			
No significant predictors. SD=3396			
**Chemotherapy – total cost model**			
Intercept	8494.87995	1661.5085	<.0001
Year	111.6406	40.52735	0.006
Age	-73.05553	22.65153	0.0013
**Radiation – total cost model**			
Intercept	5462.7329	1108.3962	<.0001
Year	69.31795	28.30658	0.0144
Age	-43.64771	14.91216	0.0035
**Chemotherapy + radiation – total cost model**			
Intercept	959.16759	1788.7597	0.5918
Year	531.2286	236.20483	0.0246
Age	22.77697	24.73902	0.3573
Year*age interaction	-7.20547	3.24035	0.0262

Parameter	Parameter Estimate	Standard error	P-value
**Best supportive care – cancer attributable cost**			
No significant predictors. SD=3614			
**Chemotherapy – cancer attributable cost model**			
Intercept	8778.4485	1674.4618	<.0001
Year	106.96173	40.8433	0.009
Age	-82.51379	22.82812	0.0003
**Radiation – cancer attributable cost**			
No significant predictors. SD=4194			
**Chemotherapy + radiation – cancer attributable cost model**			
Intercept	4083.6542	803.92827	<.0001
Age	-24.93408	11.03601	0.0239

Parameter	Parameter Estimate	Standard error	P-value
**Best supportive care – patient liability cost model**			
Intercept	893.80226	147.9069	<.0001
Age	-7.64277	1.96143	0.0001
**Chemotherapy – patient liability cost model**			
Intercept	1644.13677	279.228	<.0001
Age	-14.39425	3.7966	0.0002
**Radiation – patient liability cost model**			
Intercept	1040.04627	148.6809	<.0001
Age	-9.36982	1.96686	<.0001
**Chemotherapy + radiation – patient liability cost**			
Intercept	991.06495	111.6905	<.0001
Age	-8.44028	1.53324	<.0001

Linear regression models – continued

CONTINUING PHASE: Extensive Stage SCLC

Parameter	Parameter Estimate	Standard error	P-value
**Best supportive care – total cost**			
No significant predictors. SD=3134			
**Chemotherapy – total cost**			
No significant predictors. SD=3790			
**Chemotherapy + radiation – total cost model**			
Intercept	7279.22214	1195.4421	<.0001
Year	74.64834	27.40092	0.0065
Age	-51.12285	16.52517	0.002

Parameter	Parameter Estimate	Standard error	P-value
**Best supportive care – cancer attributable cost**			
No significant predictors. SD=3626			
**Chemotherapy – cancer attributable cost**			
No significant predictors. SD=4040			
**Chemotherapy + radiation – cancer attributable cost model**			
Intercept	7281.5117	1223.7017	<.0001
Year	59.87339	28.04866	0.0329
Age	-55.07376	16.91582	0.0011

Parameter	Parameter Estimate	Standard error	P-value
**Best supportive care – patient liability cost model**			
Intercept	-559.21914	380.9801	0.1435
Age	10.06246	4.97784	0.0444
**Chemotherapy – patient liability cost model**			
Intercept	1388.25038	332.699	<.0001
Age	-10.58672	4.52229	0.0196
**Chemotherapy + radiation – patient liability cost model**			
Intercept	1381.47033	201.488	<.0001
Age	-9.67808	2.78571	0.0005

Linear regression models – continued

TERMINAL PHASE

Parameter	Parameter Estimate	Standard error	P-value
**Terminal phase total cost – lung cancer death model**			
Intercept	24436	1000.5904	<.0001
Year	411.76653	141.68309	0.0037
Age	-148.32874	12.96255	<.0001
Age*year interaction	-6.77816	1.82769	0.0002
**Terminal phase patient liability cost – lung cancer death model**			
Intercept	2625.05227	71.40445	<.0001
Year	17.92041	10.11083	0.0763
Age	-18.25415	0.92504	<.0001
Age*year interaction	-0.41223	0.13043	0.0016
**Terminal phase total cost – other cause of death model**			
Intercept	38642	1177.6066	<.0001
Year	-510.48039	26.45033	<.0001
Age	-281.39361	14.9564	<.0001
**Terminal phase patient liability cost – other cause of death model**			
Intercept	2446.45154	163.21008	<.0001
Year	16.72101	19.28395	0.3859
Age	-14.62954	2.07771	<.0001
Year*Age interaction	-0.66203	0.24331	0.0065

Linear regression models – **Medicare Part D**

PREDIAGNOSIS PHASE

Parameter	Parameter Estimate	Standard error	P-value
**Prediagnosis phase total cost model**			
Intercept	-174.346	6.88136	<.0001
Year	48.56909	0.82392	<.0001
**Prediagnosis phase patient liability cost model**			
Intercept	-24.1497	21.73472	0.2665
Age	-0.07813	0.29075	0.7882
Year	1.64319	2.55301	0.5198
Age*year interaction	0.09679	0.03405	0.0045

SURGERY PHASE

Parameter	Parameter Estimate	Standard error	P-value
**Surgery phase total cost model**			
Intercept	186.4278	79.34285	0.0188
Age	-3.83333	1.06231	0.0003
Year	40.34639	1.79771	<.0001
**Surgery phase patient liability cost model**			
Intercept	40.09406	59.17966	0.4981
Age	-0.87332	0.79666	0.273
Year	-7.22134	8.33104	0.3861
Age*year interaction	0.23158	0.11173	0.0383

Linear regression models – **Medicare Part D**

STAGING PHASE

Parameter	Parameter Estimate	Standard error	P-value
**Staging phase – total cost model**			
Intercept	-784.173	275.9306	0.0045
Age	8.1012	3.6684	0.0272
Year	175.8657	33.01341	<.0001
Age*year interaction	-1.49603	0.43824	0.0006
**Staging phase – patient cost model**			
Intercept	-201.584	82.62285	0.0147
Age	2.3521	1.09844	0.0323
Year	33.11238	9.88532	0.0008
Age*year interaction	-0.28757	0.13122	0.0284

Linear regression models – **Medicare Part D**

INITIAL PHASE

Parameter	Parameter Estimate	Standard error	P-value
**Initial phase – total cost model**			
Intercept	-203.852	75.45247	0.0069
Age	2.38939	0.99584	0.0164
Year	34.69896	2.17642	<.0001
**Initial phase – patient cost model**			
Intercept	-26.0154	16.41424	0.113
Age	0.52295	0.21664	0.0158
Year	3.46622	0.47347	<.0001

Parameter	Parameter Estimate	Standard error	P-value
**Best supportive care – cancer attributable cost model**			
Intercept	-956.587	278.0509	0.0006
Age	11.20547	3.5652	0.0017
Year	59.85245	9.48675	<.0001
**Surgery – cancer attributable cost model**			
Intercept	-7.64912	12.48877	0.5402
Year	13.45974	1.80028	<.0001
**Chemotherapy – cancer attributable cost model**			
Intercept	-125.95	100.4879	0.2103
Year	58.84795	12.46009	<.0001
**Radiation – cancer attributable cost model**			
Intercept	27.99345	58.18268	0.6305
Year	26.99789	6.9223	<.0001
**Chemotherapy + radiation – cancer attributable cost model**			
Intercept	-2.70958	46.51481	0.9535
Year	39.84879	5.52671	<.0001

Linear regression models – **Medicare Part D**

CONTINUING PHASE

Parameter	Parameter Estimate	Standard error	P-value
**Continuing phase – total cost model**			
Intercept	-2431.74	65.75906	<.0001
Year	224.3076	6.41873	<.0001
Age	35.78862	0.94744	<.0001
Age*year interaction	-3.16603	0.08833	<.0001
**Continuing phase –patient cost model**			
Intercept	-425.704	10.52635	<.0001
Year	34.26401	1.02748	<.0001
Age	6.13502	0.15166	<.0001
Age*year interaction	-0.49233	0.01414	<.0001

Parameter	Parameter Estimate	Standard error	P-value
**Best supportive care – cancer attributable cost model**			
Intercept	-2095.27	192.4757	<.0001
Year	153.1495	19.96528	<.0001
Age	30.35047	2.75869	<.0001
Age*year interaction	-2.11181	0.27673	<.0001
**Surgery – cancer attributable cost model**			
Intercept	-2006.49	77.48267	<.0001
Year	136.4654	8.14973	<.0001
Age	29.68617	1.11573	<.0001
Age*year interaction	-1.98611	0.11202	<.0001
**Chemotherapy – cancer attributable cost model**			
Intercept	-3648.6	373.6447	<.0001
Year	364.9317	35.58666	<.0001
Age	54.37196	5.39693	<.0001
Age*year interaction	-5.11135	0.48977	<.0001
**Radiation – cancer attributable cost model**			
Intercept	-2217.87	173.6502	<.0001
Year	176.4279	17.83697	<.0001
Age	31.38004	2.50065	<.0001
Age*year interaction	-2.48072	0.24781	<.0001
**Chemotherapy + radiation – cancer attributable cost model**			
Intercept	-3381.11	196.1774	<.0001
Year	342.2106	17.62129	<.0001
Age	50.29014	2.83261	<.0001
Age*year interaction	-4.83965	0.24107	<.0001

Linear regression models – **Medicare Part D**

TERMINAL PHASE

Paramet er	Parameter Estimate	Standard error	P-value
**Terminal phase – total cost model**			
Intercept	-291.03	472.7504	0.5382
Age	9.49237	6.073	0.1181
Year	184.8391	48.42188	0.0001
Age*year interaction	-2.07166	0.62034	0.0008
**Terminal phase – patient cost model**			
Intercept	210.0956	17.53507	<.0001
Age	-0.52251	0.21186	0.0137
Year	-6.33767	0.78614	<.0001
